# Blood essential trace elements and Alzheimer’s disease biomarkers in midlife

**DOI:** 10.3389/fnagi.2025.1539749

**Published:** 2025-05-30

**Authors:** Xin Wang, Kelly M. Bakulski, Carrie A. Karvonen-Gutierrez, Sung Kyun Park, David Morgan, Brian P. Jackson, Roger L. Albin, Henry L. Paulson

**Affiliations:** ^1^Department of Epidemiology, School of Public Health, University of Michigan, Ann Arbor, MI, United States; ^2^Michigan Alzheimer’s Disease Center, University of Michigan, Ann Arbor, MI, United States; ^3^Department of Environmental Health Sciences, School of Public Health, University of Michigan, Ann Arbor, MI, United States; ^4^Department of Translational Neuroscience, College of Human Medicine, Grand Rapids Research Center, Michigan State University, Grand Rapids, MI, United States; ^5^Trace Element Analysis Laboratory, Earth Sciences, Dartmouth College, Hanover, NH, United States; ^6^Department of Neurology, University of Michigan, Ann Arbor, MI, United States; ^7^Neurology Service & GRECC, VAAAHS, Ann Arbor, MI, United States

**Keywords:** Alzheimer’s disease, amyloid-beta, tau, trace elements, biomarkers

## Abstract

**Background:**

Alzheimer’s disease (AD) is a progressive neurodegenerative disorder characterized by cognitive decline, impacting millions globally. Essential trace elements are implicated in key age-related physiologic processes but have not been fully examined with respect to AD etiology. This study investigates associations between serum levels of essential trace elements (manganese, iron, cobalt, copper, zinc, selenium, and molybdenum) and AD biomarkers (Aβ42, Aβ42/Aβ40 ratio, p-tau181, and total tau) in midlife women.

**Methods:**

This cross-sectional study included 194 midlife women (median age = 53.3 years) from the Study of Women’s Health Across the Nation, Michigan site. Serum levels of trace elements were measured using inductively coupled plasma-mass spectrometry, and AD biomarkers were quantified using single molecule array assays. Multivariable linear regression models assessed potential associations and Bayesian kernel machine regression (BKMR) was used to account for complex co-exposures and non-linear relationships.

**Results:**

In the multivariable linear regression models, a doubling of serum molybdenum level was associated with 9.4% higher Aβ42/40 ratio (95% CI: 0.8, 18.6%; *p* = 0.03), and a doubling of serum cobalt level with 17.5% higher p-tau181 level (95% CI: 3.1, 33.8%; *p* = 0.02). Copper showed an inverse association with the Aβ42/40 ratio, while zinc was positively associated with the Aβ42/40 ratio, though these associations were marginally significant. BKMR analysis confirmed these associations.

**Conclusion:**

This study identified statistically significant associations of serum molybdenum and cobalt levels with AD biomarkers, suggesting a potential protective effect of molybdenum against Aβ aggregation and exacerbation of pathologic tau phosphorylation by cobalt. These findings underscore the need for further longitudinal studies to explore the role of essential trace elements in AD pathogenesis.

## 1 Introduction

Alzheimer’s disease (AD), a progressive neurodegenerative disorder and common cause of dementia, affects millions worldwide (Hebert et al., 2013). Characterized by accumulation of amyloid-β (Aβ) plaques and neurofibrillary tangles of tau protein, AD leads to a decline in cognitive function, ultimately impairing daily life activities. Early detection and intervention are crucial for altering the course of AD, offering a window of opportunity to delay or prevent progression to overt cognitive impairment. Midlife is increasingly recognized as a crucial period for early changes in cognitive function that may lead to dementia, as research shows that molecular, cellular, and structural brain alterations—such as hippocampal shrinkage, white matter loss, and neuroinflammation—accelerate during this time, setting the stage for later cognitive decline (Deckers et al., 2015; Karlamangla et al., 2017; Dohm-Hansen et al., 2024; Won et al., 2025). Within this context, biomarkers related to the pathological hallmarks of AD have emerged as potential early predictors. Our most recent findings indicate that a lower Aβ42/40 ratio and higher phosphorylated tau181 (p-tau181) levels in midlife women are associated with accelerated cognitive decline from mid-to late life (Wang et al., 2024). These results underscore the potential of these biomarkers in signaling early changes, identifying potential risk factors, and understanding mechanisms of neurodegeneration.

The role of essential trace elements in AD has garnered increased attention, with elements such as chromium (Cr), manganese (Mn), iron (Fe), cobalt (Co), copper (Cu), zinc (Zn), selenium (Se), and molybdenum (Mo) crucial for biological functions. Dysregulation in homeostasis of these elements is implicated in AD pathogenesis (De Benedictis et al., 2019). Cu and Zn, for example, directly bind Aβ, facilitating aggregation into insoluble fibrils and oligomers, potentially promoting development of senile plaques (Bush, 2013; Squitti et al., 2014). Fe is implicated in hyperphosphorylation of tau protein, leading to neurofibrillary tangle formation (Derry et al., 2020). Mn and Se are crucial in modulating oxidative stress, and alterations in their homeostasis can exacerbate oxidative damage and neuroinflammation (Takeda, 2003; Cardoso et al., 2015). With the exception of Se, human studies examining the potential effects of essential trace elements are relatively limited and have focused on cognitive correlates (Cheng et al., 2022; Shang et al., 2021; Gu et al., 2021). Lower levels of Se were found in AD patients compared to control groups, suggesting a potential protective role of Se against AD pathogenesis (Varikasuvu et al., 2019; Loef et al., 2011). The majority of prior research focused on older populations and to date, no study has investigated potential associations between essential trace elements and AD biomarkers in midlife.

To address this research gap, we investigated the associations between serum levels of essential trace elements (Mn, Fe, Co, Cu, Zn, Se, and Mo) and serum levels of AD biomarkers (Aβ42, the Aβ42/Aβ40 ratio, p-tau181, and total tau) in 189 midlife women from the Study of Women’s Health Across the Nation (SWAN) at the Michigan site.

## 2 Materials and methods

### 2.1 Study population

SWAN is an ongoing, multi-racial/ethnic, community-based study initiated in 1996–1997 to evaluate the menopausal transition and its physical and psychological impacts. The study recruited 3,302 premenopausal women aged 42–52 from seven locations across the United States. Eligibility criteria included an intact uterus and at least one ovary, menses in the last 3 months, and not using hormone therapy in the previous 3 months (Santoro et al., 2011). A full description of the study design is available (Wang et al., 2022). Institutional Review Board approval was obtained at each study site of SWAN, and all participants provided signed informed consent at each study visit. All methods were performed in accordance with relevant guidelines and regulations and followed the Strengthening the Reporting of Observational Studies for Epidemiology (STROBE) guidelines.

**Figure 1:**
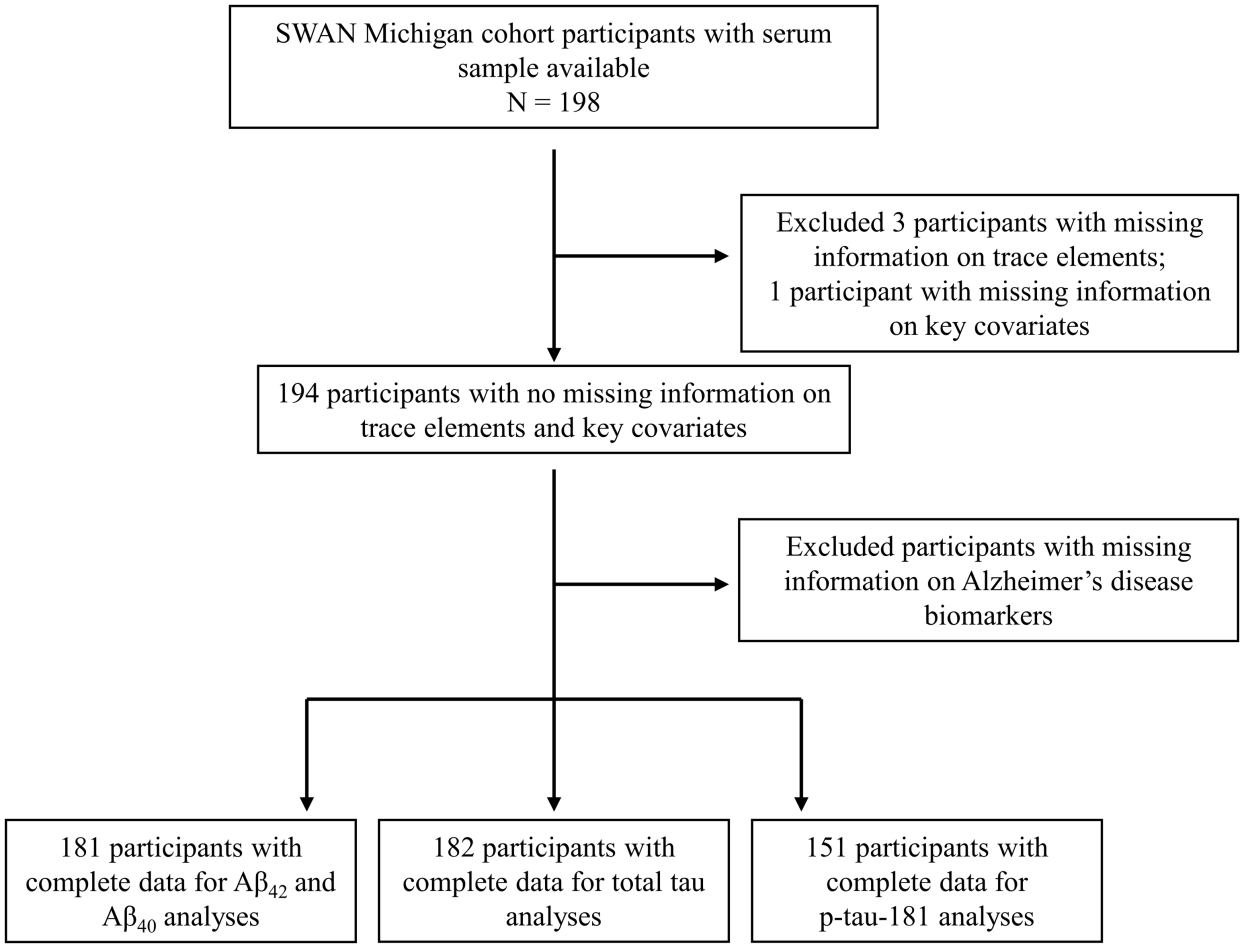
Flow chart of the study design.

The current analysis included data from 198 women at the Michigan SWAN site with available serum samples collected in 2003–2004. Exclusion criteria for the analytic sample were applied as follows: three participants excluded due to inadequate serum volume; one participant excluded for lack of covariate information; 13 excluded for missing Aβ42 and Aβ40 data; 12 for absent total tau data; and 43 for missing p-tau181. Consequently, the final analytical sample includes 181 participants for the Aβ42 and Aβ42/Aβ40 ratio analyses, 182 participants for the total tau analysis, and 151 participants for the p-tau181 analysis. The selection process and exclusion criteria are presented in Figure 1.

### 2.2 Serum AD biomarkers

Fasted blood samples were obtained before 10:00 am. Samples were aliquoted and preserved at −80°C in the SWAN Repository until transport in their frozen state to the Biomarker Core Lab of the Michigan Alzheimer’s Disease Research Center at Michigan State University. Serum levels of Aβ42, Aβ40, total tau, and p-tau181 were determined using Single Molecule Array (Simoa) assays (Quanterix in Billerica, MA, United States). For quality control, each assay included analysis of duplicate samples, with a predefined acceptable coefficient of variation set below 15% for all biomarkers. Pooled serum bridge samples were integrated into each assay batch and no variations were found between assays (Wang et al., 2024).

### 2.3 Serum essential trace elements

Serum levels of essential trace elements, including Mn, Fe, Co, Cu, Zn, Se, and Mo, were analyzed with triple quadrupole inductively coupled plasma-mass spectrometry (ICP-MS) at the Dartmouth College Trace Element Analysis Core. Quality control measures including continuous calibration verification, analysis of duplicates and spiked samples, intra-and inter-batch analyses, and comparison against certified reference materials, rigorously applied in line with EPA SW-846 Quality Control standards (Seronorm Serum levels 1 and 2, Biilingstad, Norway) and Methodology 6020B (US EPA, 2015). Mean recoveries of reference materials (n = 21) for Mn, Fe, Co, Cu, Zn, Se, and Mo were: 107 ± 9%, 105 ± 6%, 110 ± 12%, 101 ± 4%, 94 ± 4%, 91 ± 5%, and 111 ± 11%, respectively.

### 2.4 Covariates

Demographic information including age, self-identified race (Black or White), and education (categorized into high school or lower, some college, or a college degree and above) was collected through self-administered questionnaires. Smoking status was defined as never smoker, former smoker, and current smoker. The frequency of alcohol intake was categorized into less than one drink per month, one drink per month to one drink per week, and more than one drink per week. Menopausal status (pre-menopausal, early peri-menopausal, late peri-menopausal, post-menopausal, or indeterminable due to hormone therapy usage or hysterectomy) was evaluated through standardized interviews about bleeding patterns and exogenous hormone use.

### 2.5 Statistical analysis

We described the distributions of covariates using number and frequency for categorical variables and median and interquartile range (IQR) for continuous covariates. For element distributions, we additionally reported the LOD, frequency of observations above the LOD, and the 10th and 90th percentiles. We used multivariable linear regression models to evaluate the potential associations between levels of essential trace elements and AD biomarkers. Given the right-skewed distribution of both the AD biomarkers and trace element levels, we applied logarithmic transformations so that shapes of exposure-outcome relationships more closely approximated log-linear. Specifically, natural logarithms were used for the AD biomarkers, and base two logarithms were applied to the trace element levels. Thus, the associations were interpreted as the percent difference in AD biomarkers per doubling of each essential trace element level. The covariates adjusted in the models were based on a prior knowledge and included age, race, education, smoking status, alcohol drinking, and menopausal status (Wang et al., 2024; Wang et al., 2019).

In the secondary analysis, we employed the Bayesian Kernel Machine Regression (BKMR) (Bobb et al., 2015) to account for the complexities of co-exposure to various trace elements and the potential for non-linear relationships between these elements and AD biomarkers. More specifically, BKMR allows the flexible exposure-response functions for each essential trace element in relation to AD biomarkers, while maintaining all other trace element levels at their median values. Gaussian kernel exposure response machine function was used to fit the model. The analysis adjusted for the same covariates as in the linear regression models to ensure consistency. The BKMR analysis was performed using the ‘bkmr’ package (Bobb et al., 2015) and all analyses were conducted using R, version 4.3.1.^1^

## 3 Results

### 3.1 Sample descriptive statistics

The median (interquartile range, IQR) age of the analytic sample was 53.3 (51.0, 55.6) years and 61% of participants identified as Black. Most participants were never smokers, reported alcohol consumption less than once monthly, and were post-menopausal (Table 1). Details regarding the distribution of essential trace elements, including their LODs and detection rates, are provided in Supplementary Table S1. Notably, all elements exhibited a detection rate of 100%.

**Table 1:** Characteristics of the study population with at least one Alzheimer’s disease biomarker (*N* = 189).

Characteristics	Distribution
Age, years, median (IQR)	53.3 (51.0, 55.6)
Race, *n* (%)	
White	73 (38.6)
Black	116 (61.4)
Education, *n* (%)	
High school or less	128 (67.7)
Some college	29 (15.3)
College and above	32 (16.9)
Smoking status, *n* (%)	
Never smoked	104 (55.0)
Former smoker	50 (26.5)
Current smoker	35 (18.5)
Alcohol drinking, *n* (%)	
≤ 1 drink/month	121 (64.0)
> 1 drink/month and ≤1/week	38 (20.1)
> 1 drink/week	30 (15.9)
Menopausal status, *n* (%)	
Pre-menopausal	2 (1.1)
Early peri-menopausal	51 (27.0)
Late peri-menopausal	23 (12.2)
Post-menopausal	100 (52.9)
Unknown^a^	13 (7.9)
Chromium, μg/L, median (IQR)	0.87 (0.77, 0.97)
Manganese, μg/L, median (IQR)	0.81 (0.68, 1.02)
Iron, μg/L, median (IQR)	1,146 (926, 1,590)
Cobalt, μg/L, median (IQR)	0.11 (0.09, 0.16)
Copper, μg/L, median (IQR)	1,259 (1,094, 1,398)
Zinc, μg/L, median (IQR)	742 (697, 813)
Selenium, μg/L, median (IQR)	117.4 (108.4, 126.7)
Molybdenum, μg/L, median (IQR)	0.94 (0.77, 1.19)
Aβ42, pg./mL, median (IQR)	8.3 (4.2, 11.1)
Aβ40, pg./mL, median (IQR)	174.9 (82.0, 219.2)
Aβ42/40 ratio, median (IQR)	0.05 (0.04, 0.06)
Total tau, pg./mL, median (IQR)	2.7 (1.5, 4.5)
Phosphorylated tau181, median (IQR)	14.3 (9.8, 19.3)

IQR, interquartile range.

a Menopausal status unknown due to hormone therapy or hysterectomy.

### 3.2 Associations between trace elements and AD biomarkers using linear regression

In linear regression models, after adjustment for age, race, education, smoking status, alcohol drinking, and menopausal status, each doubling of serum Mo was associated with 9.4% (95% CI: 0.8, 18.6%; *p* = 0.03) higher Aβ42/40 ratio (Table 2). Serum Co was statistically significantly associated with p-tau181; doubling of Co levels was associated with 17.5% (95% CI: 3.1, 33.8%; *p* = 0.02) higher p-tau181 levels. Associations were observed for serum Cu with −15.1% lower Aβ42/40 ratio (95% CI: −29.3, 1.9%; *p* = 0.08), and serum Zn with 35.1% higher (95% CI: −0.5, 83.3%; *p* = 0.05) for each doubling of the trace elements, respectively, though the associations were borderline statistically significant. No significant or suggestive associations were found between essential trace elements and Aβ42 levels.

**Table 2:** Associations between serum Alzheimer’s disease (AD) biomarkers and essential trace elements in multivariable-adjusted linear regressions.

Elements	Aβ42		Aβ42/40 ratio		Total tau		p-tau181	
	Percentage change (95% CI)^a^	*p*-value	Percentage change (95% CI)^a^	*p*-value	Percentage change (95% CI)^a^	*p*-value	Percentage change (95% CI)^a^	*p*-value
Cr	30.2 (−6.9, 82.0)	0.12	12.9 (−2.8, 31.1)	0.11	2.8 (−28.5, 47.9)	0.88	16.8 (−15.6, 61.5)	0.35
Mn	7.2 (−9.1, 26.5)	0.41	3.3 (−4.1, 11.3)	0.39	5.9 (−11.6, 26.9)	0.53	6.8 (−7.1, 22.8)	0.35
Fe	1.2 (−13.6, 18.5)	0.88	−0.5 (−7.3, 6.9)	0.90	15.4 (−2.7, 36.9)	0.09	8.5 (−4.0, 22.8)	0.19
Co	11.9 (−4.2, 30.7)	0.15	0.1 (−6.7, 7.4)	0.98	0.5 (−14.9, 18.6)	0.96	17.5 (3.1, 33.8)	0.02
Cu	14.0 (−24.6, 72.2)	0.53	−15.1 (−29.3, 1.9)	0.08	4.6 (−33.3, 64.0)	0.84	−10.4 (−35.6, 24.7)	0.51
Zn	−4.6 (−52.4, 91.1)	0.89	35.1 (−0.5, 83.3)	0.05	−28.1 (−66.3, 53.2)	0.39	−24.0 (−56.7, 33.5)	0.34
Se	−8.8 (−49.1, 63.6)	0.76	19.1 (−8.3, 54.7)	0.19	−41.9 (−69.1, 9.1)	0.09	−1.1 (−45.1, 78.5)	0.97
Mo	−2.3 (−18.8, 17.6)	0.80	9.4 (0.8, 18.6)	0.03	0.4 (−18.1, 22.9)	0.97	−2.9 (−16.7, 13.2)	0.70

LOD, limit of detection; Cr, chromium; Mn, manganese; Fe, iron; Co, cobalt; Cu, copper; Se, selenium; Mo, molybdenum; p-tau181, phosphorylated tau181.

a Levels of AD biomarkers were log-transformed and essential trace elements were log2-transformed. Thus, associations were interpreted as the percent changes in AD biomarkers per doubling of each essential trace element level. All regression models were adjusted for age, race, education, smoking status, alcohol drinking, and menopausal status.

### 3.3 Associations between trace elements and AD biomarkers using BKMR

**Figure 2:**
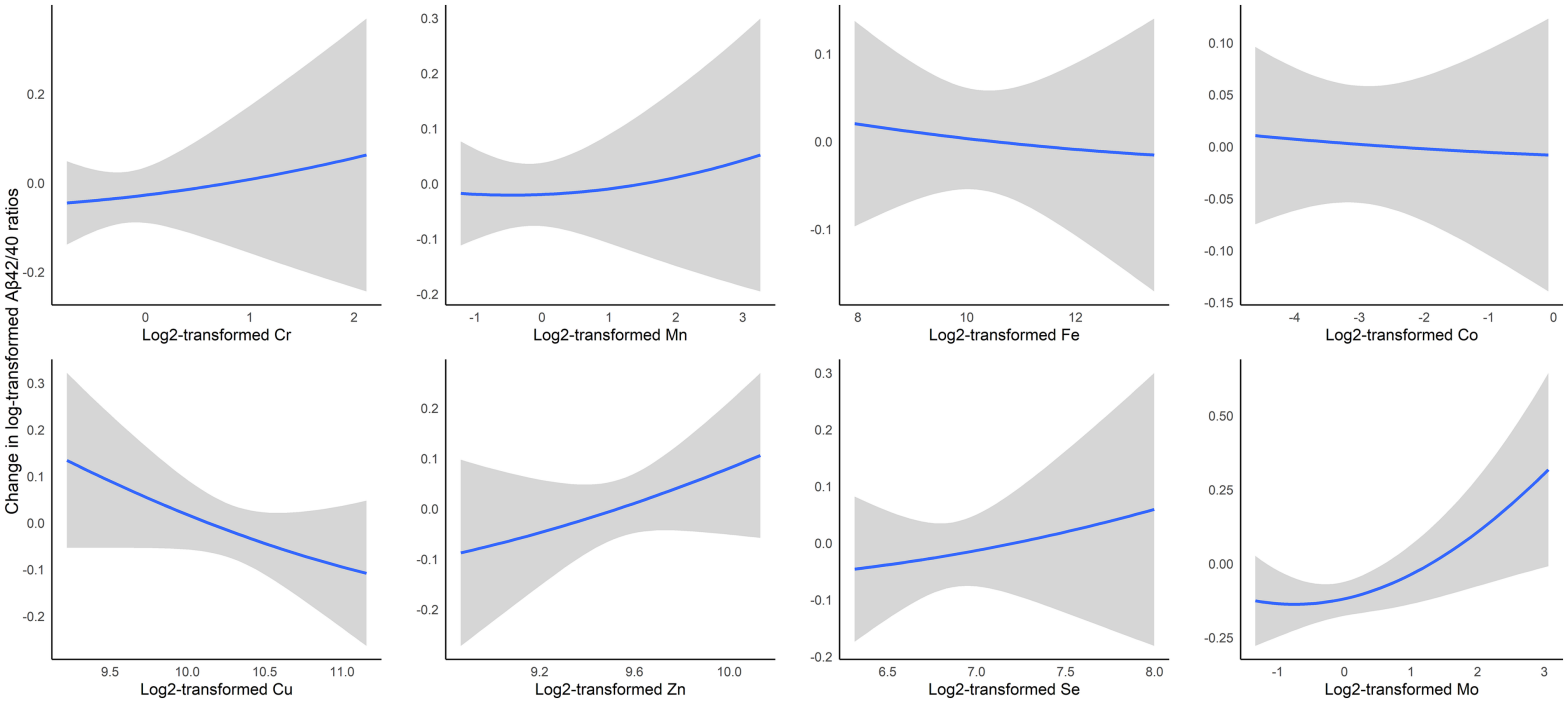
Exposure-outcome relationships and 95% confidence interval (95% CI) bands for each essential trace element with Aβ42/40 ratios while holding all other elements at median levels, estimated by Bayesian kernel machine regression. The model was adjusted for age, race, education, smoking status, alcohol drinking, and menopausal status. Cr, chromium; Mn, manganese; Fe, iron; Co, cobalt; Cu, copper; Se, selenium; Mo, molybdenum.

In our secondary analysis, we employed BKMR to simultaneously incorporate all eight trace elements into the model, with each AD biomarker serving as the outcome. This comprehensive approach revealed specific association patterns presented in Figure 2 and Supplementary Figures S1–S3. Notably, Mo and Zn showed positive associations with the Aβ42/40 ratio, and Cu was inversely related (Figure 2). For total tau levels, Fe was positively associated, whereas Se had an inverse relationship (Supplementary Figure S2). In the case of p-tau181, Co exhibited a positive association, and Zn showed an inverse association (Supplementary Figure S3). These associations generally followed a log-linear pattern. Like in the linear regression analyses, no associations were found between essential trace elements and Aβ42 (Supplementary Figure S1).

## 4 Discussion

This study revealed associations between serum levels of essential trace elements and AD biomarkers in midlife women. We found that higher levels of Mo were associated with higher Aβ42/40 ratio, and elevated Co levels associated positively with p-tau181 levels. Additionally, Cu showed an inverse association with the Aβ42/40 ratio, while Zn was positively associated with Aβ42/40 respectively, though associations were borderline statistically significant.

Our findings regarding the association between higher Mo and higher Aβ42/40 ratio might indicate a potential protective role of Mo against Aβ aggregation. Aβ42 is more prone to aggregation and plaque formation than Aβ40, and thus, a lower Aβ42/40 ratio is typically indicative of a shift toward a pathological state in which amyloid plaques are more likely to form in the brain (Hampel et al., 2021). This shift has been associated with accelerated cognitive decline and AD progression (Wang et al., 2024; Giudici et al., 2020; Graff-Radford et al., 2007). Notably, the Aβ42/40 ratio is considered a more sensitive marker of amyloid pathology than Aβ42 alone. Mo deficiency is rare (Novotny, 2011), and the Mo levels in our study were within the range of a previous study of healthy women (Versieck et al., 1978). An essential cofactor for enzymes including sulfite oxidase, aldehyde oxidase, and xanthine oxidase, Mo is pivotal in biological mechanisms including oxidative stress regulation, purine metabolism, and potential modulation of Aβ dynamics (Botchway et al., 2023). By facilitating the activity of antioxidant enzymes, Mo might mitigate oxidative damage to neurons (Wang et al., 2014) and influence uric acid levels (Coelho et al., 2022), a correlate of reduced risk of AD and cognitive decline (Scheepers et al., 2019; Liu et al., 2017). Thus, several potential mechanisms exist for which Mo could impact AD progression. Recent experimental model work highlighted the potential of Mo-containing compounds to directly interfere with Aβ aggregation and promote Aβ clearance (Han et al., 2019). There is a notable scarcity of human studies specifically examining the association of Mo with AD or its biomarkers. A few studies explored the relationship between Mo levels and cognitive functions, with mixed results (Smorgon et al., 2004; Xiao et al., 2021). Another study comparing trace element concentrations demonstrated that serum molybdenum levels were significantly lower in patients with AD or mild cognitive impairment compared to individuals with subjective memory complaints and cognitively normal controls (Paglia et al., 2016).

The observed association between higher Co levels and elevated p-tau181 levels in our study, with serum Co levels in the normal range (Chen and Lee, 2024), suggests a potential neurotoxic pathway that may contribute to the pathogenesis of AD. Co, vital for vitamin B12 synthesis, is implicated in neurodegeneration through pathways outside its traditional nutritional roles (Jatoi et al., 2020). This is highlighted by potential links between Co levels and Peptidyl-prolyl cis–trans isomerase NIMA-interacting 1 (PIN-1) expression. Reduced PIN-1 activity is implicated in pathologic processing of tau and amyloid precursor protein (APP). A recent murine model study demonstrated that Co exposure decreases PIN-1 expression, leading to cell cycle arrest and apoptosis in neuroglioma cells (Zheng et al., 2021). Elevated blood Co levels were associated with disrupted PIN-1 activity, increased tau phosphorylation, and neuronal loss in cerebral cortex and hippocampus. The same research group showed an inversed association between blood Co and PIN-1 levels in 30 patients with cobalt alloy hip replacements, supporting findings from animal studies (Zheng et al., 2021). Additionally, exposure to Co in mice has been shown to induce tau hyperphosphorylation, Aβ deposition, and dysregulated autophagy in the hippocampus and cortex, mediated by an increase in ROS production through the activation of hypoxia-inducible factor-1α (Tang et al., 2023). Direct research in humans investigating the cobalt-AD linkage is limited. A recent study involving over 6,000 participants with a mean age of 62 years at baseline found that baseline urinary Co concentration was associated with lower cognitive performance measured 10 years later, especially among *APOE*4 carriers (Domingo-Relloso et al., 2024).

Our research provides evidence for potential associations of Cu and Zn with AD biomarkers, though these findings are of marginal statistical significance. These trace elements are implicated in various neurological functions and their dysregulation may contribute to the pathogenesis of AD. Cu is crucial for brain functions such as oxygen transport, neurotransmitter synthesis, and energy metabolism (Botchway et al., 2023). Dysregulated Cu homeostasis is potentially associated with AD pathology through several mechanisms. Elevated levels of ceruloplasmin, a Cu-binding protein, in AD patients suggest altered Cu homeostasis (Wang et al., 2015; Squitti, 2012). However, ceruloplasmin is generally and non-specifically elevated in inflammatory states, which may complicate its role as a specific marker for AD-related Cu dysregulation. The role of Cu in glutamatergic neurotransmission is also linked to the glutamatergic dysfunction observed in AD (Zheng et al., 2010). Additionally, Cu may directly interact with APP, affecting amyloidogenic processing and promoting the synthesis of Aβ peptides (Jacobsen and Iverfeldt, 2009; Thinakaran and Koo, 2008). However, the therapeutic use of copper chelators in AD patients has not yielded significant results, indicating that targeting Cu through chelation alone may not be sufficient to alter disease progression (Gromadzka et al., 2020). Zn is essential for metalloenzyme activity, neurotransmission, neurogenesis, and cognitive functions such as learning and memory. In AD, disruptions in Zn homeostasis, particularly the inhibited expression of Zn transporters, may contribute to disease progression (Xu et al., 2019). Evidence on Zn supplementation is mixed. While some animal studies have reported adverse effects of Zn on Aβ and APP, which further linked to compromised memory and spatial learning (Wang et al., 2010; Yang et al., 2013), Zn supplementation has shown promise in improving cognitive function, reducing Aβ and tau pathologies, and regulating oxidative stress in other animal studies and clinical trials (Corona et al., 2010; Sandusky-Beltran et al., 2017; Hosseini et al., 2021). Together, these findings highlight the complex roles of Cu and Zn in AD and underscore the need for further research using brain-specific measures and mechanistic studies to clarify their potential as targets for intervention.

To our knowledge, this is the first investigation of relationships between essential trace elements and AD biomarkers in midlife adults. Our findings offer important considerations for approaches to AD prevention, perhaps including modulation of trace element levels as part of a broader strategy that includes lifestyle and dietary interventions. However, our findings are limited by the modest sample size drawn from the SWAN Michigan cohort, which impacts statistical power. Furthermore, the study population composition, excluding men and limited to Black and White women by design, narrows the generalizability of our findings. Moreover, our cross-sectional assessment of element levels and AD biomarkers were confined to a single time point. We encourage future investigations to incorporate repeated assessments of AD biomarkers to explore the relationship between trace elements and their longitudinal changes.

In summary, our study provides novel results on associations between serum levels of essential trace elements and AD biomarkers in midlife women. We identified significant associations of Mo and Co with AD biomarkers, with Mo potentially offering protective effects against Aβ aggregation and Co suggesting a neurotoxic pathway linked to tau phosphorylation. Our research also highlights the importance of Cu and Zn in AD biomarker profiles, despite marginal statistical significances, pointing to their roles in AD pathogenesis. A significant body of preclinical work supports different but potentially significant roles for essential trace elements in AD pathogenesis. Our results underscore the need for further investigations into the role of trace elements in AD, particularly through longitudinal studies and with larger, diverse cohorts, to validate our findings and explore their implications for early AD diagnosis and potential interventions. The introduction of trace element modulation as part of a comprehensive strategy for AD prevention opens promising avenues for future research and healthcare approaches in the management and prevention of AD.

## Data Availability

The data analyzed in this study is subject to the following licenses/restrictions: the data that support the findings of this study are not publicly available due to privacy concerns and ethical restrictions. Requests to access these datasets should be directed to xwangsph@umich.edu.

## Footnotes

^1^ www.R-project.org
