# Supplementary material for: Blood essential trace elements and Alzheimer’s disease biomarkers in midlife
Source: Front Aging Neurosci. 2025 May 30;17:1539749. doi: 10.3389/fnagi.2025.1539749 (PMC12162987; doi:10.3389/fnagi.2025.1539749)
Supplement: Supplementary file 1 [file Table_1.docx]

**Supplemental Materials: Blood Essential Trace Elements and Alzheimer’s Disease Biomarkers in Midlife**

Xin Wang^a^, Kelly M. Bakulski^a,b^, Carrie A. Karvonen-Gutierrez^a^, Sung Kyun Park^a,c^_,_ David Morgan^d^, Brian P. Jackson^e^, Roger L. Albin^b,f,g^_,_ Henry L. Paulson^b,f^

^a^ Department of Epidemiology, School of Public Health, University of Michigan, Ann Arbor, MI, USA, 48109.

^b^ Michigan Alzheimer’s Disease Center, University of Michigan, Ann Arbor, MI, USA, 48105.

^c^ Department of Environmental Health Sciences, School of Public Health, University of Michigan, Ann Arbor, MI, USA, 48109.

^d^ Department of Translational Neuroscience, College of Human Medicine, Grand Rapids Research Center, Michigan State University, Grand Rapids, MI, USA, 49503.

^e^ Trace Element Analysis Laboratory, Earth Sciences, Dartmouth College, Hanover, NH, 03755, USA

^f^ Department of Neurology, University of Michigan, Ann Arbor, MI, USA, 48109.

^g^ Neurology Service & GRECC, VAAAHS, Ann Arbor, MI, USA, 48105.

Correspondence to Xin Wang, Department of Epidemiology, University of Michigan, M5523 SPH II, 1415 Washington Heights, Ann Arbor, Michigan, USA, 48109-2029. Email: [xwangsph@umich.edu](mailto:xwangsph@umich.edu). ORCID: 0000-0002-0851-6605.

**Table S1.** Distributions and detection rates of serum essential trace elements.

| **Elements** | **LOD** | **Percent >LOD** | **10^th^ percentile** | **25^th^ percentile** | **Median** | **75^th^ percentile** | **90^th^ percentile** |
| --- | --- | --- | --- | --- | --- | --- | --- |
| Cr, µg/L | 0.62 | 100 | 0.71 | 0.77 | 0.87 | 0.97 | 1.16 |
| Mn, µg/L | 0.06 | 100 | 0.58 | 0.68 | 0.81 | 1.02 | 1.95 |
| Fe, µg/L | 6 | 100 | 709 | 926 | 1146 | 1590 | 2161 |
| Co, µg/L | 0.005 | 100 | 0.072 | 0.087 | 0.107 | 0.157 | 0.307 |
| Cu, µg/L | 3 | 100 | 970 | 1094 | 1259 | 1398 | 1591 |
| Zn, µg/L | 2 | 100 | 646 | 697 | 742 | 813 | 884 |
| Se, µg/L | 0.4 | 100 | 98.4 | 108.4 | 117.4 | 126.7 | 138.3 |
| Mo, µg/L | 0.08 | 100 | 0.64 | 0.77 | 0.94 | 1.19 | 1.77 |

Note: LOD: limit of detection; Cr: chromium; Mn: manganese; Fe: iron; Co: cobalt; Cu: copper; Zn: zinc; Se: selenium; Mo: molybdenum.


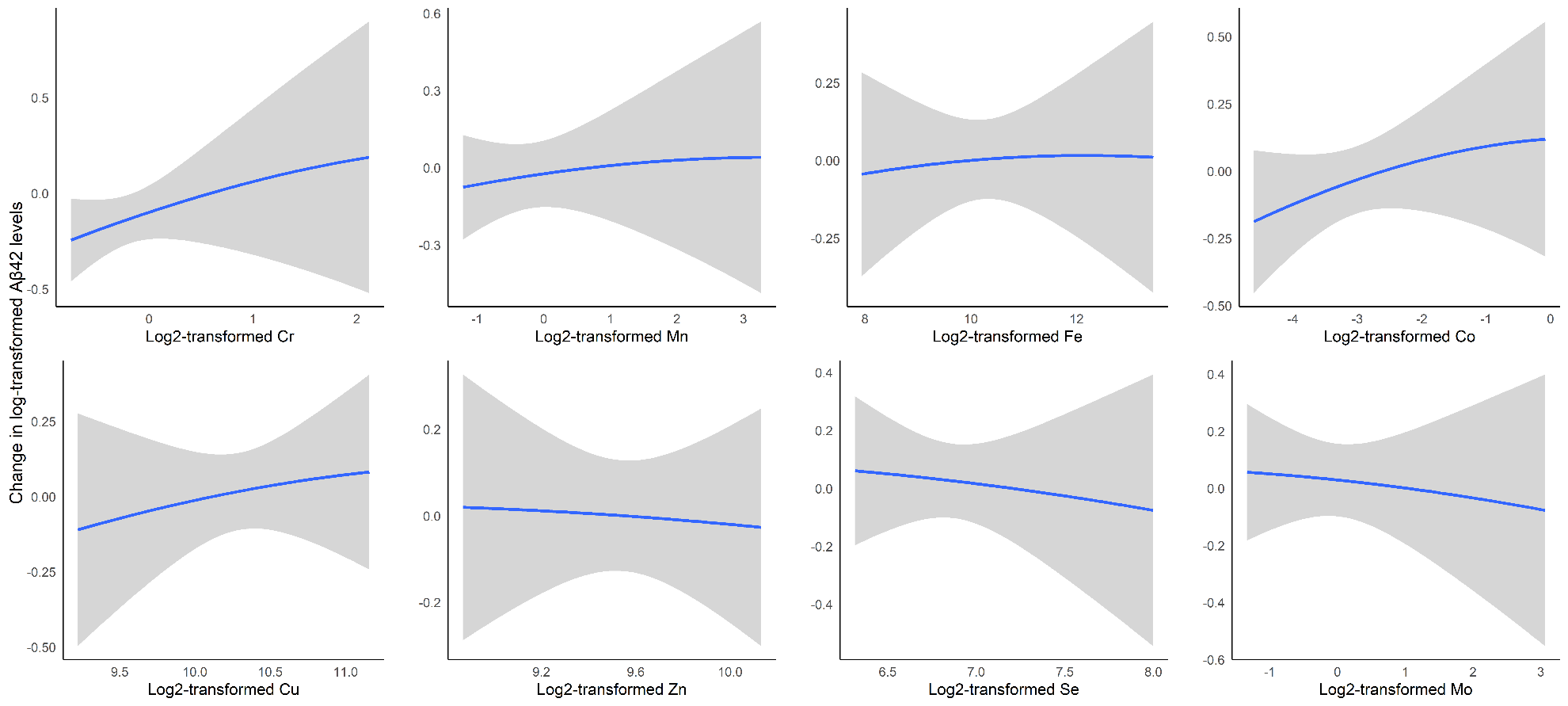


**Figure S1.** Exposure-outcome relationships and 95% confidence interval (95% CI) bands for each essential trace element with Aβ42 levels while holding all other elements at median levels, estimated by Bayesian kernel machine regression. The model was adjusted for age, race, education, smoking status, alcohol drinking, and menopausal status. Cr: chromium; Mn: manganese; Fe: iron; Co: cobalt; Cu: copper; Se: selenium; Mo: molybdenum.


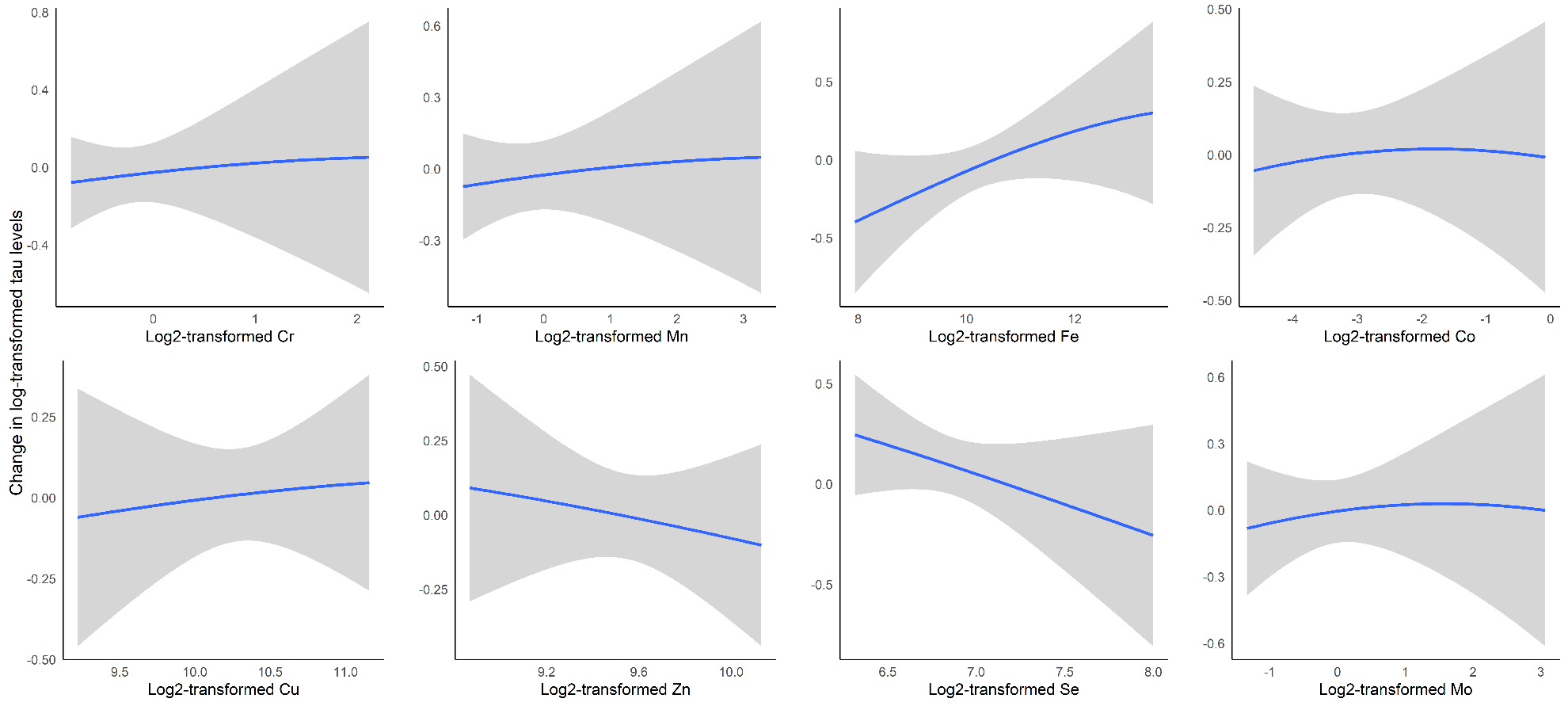


**Figure S2.** Exposure-outcome relationships and 95% confidence interval (95% CI) bands for each essential trace element with total tau levels while holding all other elements at median levels, estimated by Bayesian kernel machine regression. The model was adjusted for age, race, education, smoking status, alcohol drinking, and menopausal status. Cr: chromium; Mn: manganese; Fe: iron; Co: cobalt; Cu: copper; Se: selenium; Mo: molybdenum.


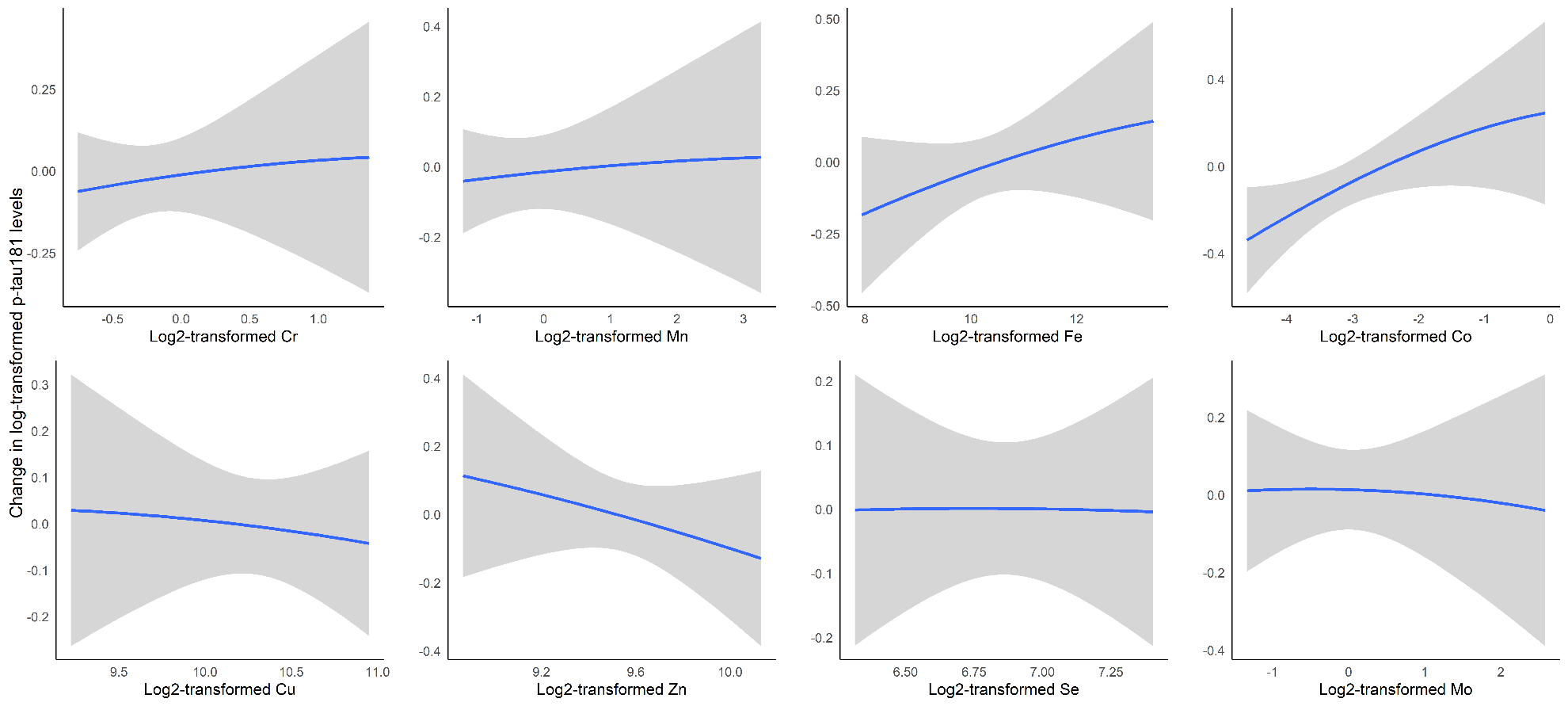


**Figure S3.** Exposure-outcome relationships and 95% confidence interval (95% CI) bands for each essential trace element with phosphorylated tau181 (p-tau181) levels while holding all other elements at median levels, estimated by Bayesian kernel machine regression. The model was adjusted for age, race, education, smoking status, alcohol drinking, and menopausal status. Cr: chromium; Mn: manganese; Fe: iron; Co: cobalt; Cu: copper; Se: selenium; Mo: molybdenum.
